# Editor's Letter-Box

**Published:** 1902-05-24

**Authors:** 


### EDITOR'S LETTER-BOX.

[Our Correspondents are reminded that prolixity is a great bar to publication, and that brevity of style and conciseness of statement greatly facilitate early insertion.]

W. G. Black, F.R.C.S.E., writes:—A very interesting article appeared in the *Edinburgh Medical Journal* for April, on fatal cases of latent pneumonia, and one appreciated the statistical tables of the cases, for their arrangement, and details in relation to intoxication.

Some two years since, on the outbreak of the beer poisoning cases at Manchester, I visited the infirmary there to see the patients, and was shown that the symptoms and fatalities were due to arsenic contained in that intoxicant. It seems to me then that these cases of fatal latent pneumonia might probably come under much the same category here as in Manchester, and might be result of poisonous whisky imbibed by the victims of intoxication, and not the result of pure alcoholism simply. About three months ago a full detailed article appeared in the *Scotsman* of the analysis of modern new whisky, which showed that it consisted, not of pure grain spirit, but of a mixture of aldehydes, fusel oil, ferfusal, naphtha, and beryl.

This concoction could scarcely be termed Scotch whisky, but more appropriately German whisky, and may therefore prove deleterious to the native stomach and constitution, and may have other poisonous ingredients in it yet undiscovered

It might therefore be suggested that in future well authenticated fatal cases the whisky that had been used as a regular intoxicant by the party might be got and analysed for the deleterious ingredients suspected.

It is of common repute that the modern whisky used by the sluming classes now tends to incite to violence and rioting, and it may therefore be well imagined that this effect might be due to irritant poisons contained in it from its course of manufacture.

One peculiarity of this latent pneumonia about Edinburgh seems to consist in its ingravescent character, the gradual invasion of consolidation of the lungs, without reflex cough, and dyspnœa or rusty expectoration, till the circulation comes to a stop, and a fatal issue takes place by collapse.

It will be seen on reference to medical text-books that it differs on most points from the description of the typical pneumonia, say of "Hooper's Physician's Vade Mecum," p. 486, so that its causation is probably quite different also.

It may then be gathered that this peculiarity is dependent more on internal *materies morbi* than on external impressions of cold or bad air on the surface of the body, and that adulterated whisky would be as powerful an agent as septic infection.

